# The cellular logic of limb regeneration

**DOI:** 10.7554/eLife.110316

**Published:** 2026-01-22

**Authors:** Matthew Cherubino, Catherine D McCusker

**Affiliations:** 1 Department of Biology, University of Massachusetts Boston Boston United States

**Keywords:** axolotl, *Ambystoma mexicanum*, limb regeneration, dorsoventral, Other

## Abstract

Hierarchical communication between cells with distinct positional memories orchestrates the regeneration of missing limb structures in axolotls.

**Related research article** Yamamoto S, Furukawa S, Ohashi A, Hamada M, Satoh A. 2025. Dorsoventral-mediated Shh induction is required for axolotl limb regeneration. *eLife* **14**:RP106917. doi: 10.7554/eLife.106917.

Human limbs are marvels of biological engineering. Bones, muscles, nerves, blood vessels and skin must be precisely organized and seamlessly interconnected for even the simplest movement to occur. This remarkable complexity is not assembled by chance: it is guided by a molecular blueprint written into limb cells during early embryonic development, when waves of signaling molecules instruct cells where they are, what they should become, and how they should connect.

In humans, however, this developmental symphony plays only once. After limb loss or severe injury, adult human cells cannot replay the signals needed to rebuild a functional limb – a limitation that makes limb loss profoundly debilitating. Other animals face no such constraint. Salamanders, such as axolotls, can regenerate complete, fully patterned limbs throughout their lives. How do their injuries succeed where ours fail?

The answer lies in a remarkable cellular memory. Salamander limb cells retain information about their original position within the limb (front versus back, top versus bottom) and use this information to communicate with one another after injury. Previous studies revealed that successful limb regeneration requires the presence of cells from both the anterior (front) and posterior (back) sides of the limb (Bryant et al., 1981; Endo et al., 2004), which together generate key organizing signals needed to rebuild missing structures from temporary organs called blastemas (Nacu et al., 2016; Vieira et al., 2019; Otsuki et al., 2025).

Yet a fundamental question has remained unresolved: do these anterior–posterior interactions operate independently, or do they rely on additional positional cues from the dorsal (top) and ventral (bottom) sides of the limb? Now, in eLife, Akira Satoh and colleagues at Okayama University – including Sakiya Yamamoto as first author – report the results of experiments which show how cells from all sides of the limb axes work together to generate the positional cues needed to regenerate limb structures (Yamamoto et al., 2025).

Yamamoto et al. worked with axolotl cells, which allows researchers to build regenerating limb tissues with carefully controlled cellular inputs. They systematically generated blastemas that were missing cells from one side of the limb at a time. As expected from earlier work (Endo et al., 2004; Nacu et al., 2016), blastemas lacking either anterior or posterior cells failed to regenerate a complete limb.

Strikingly, the researchers found that the same was true when blastemas lacked cells from either the dorsal or ventral side. This observation led them to focus on a gene called *Shh* (for Sonic hedgehog), which is known to have a central role in front–back limb patterning. They discovered that this gene was only activated when dorsal and ventral cells came into contact within the blastema. By tracing the fate of labeled cells and selectively adding or removing tissues, Yamamoto et al. showed that posterior cells could express Shh, but only when they were exposed to signals produced through dorsal-ventral interactions.

To identify these signals, the researchers compared gene expression patterns in blastemas derived primarily from dorsal cells with those derived from ventral cells. This analysis revealed two key molecules involved in cell signaling and growth: WNT10B, which was enriched in dorsal-derived blastemas, and FGF2, which was enriched in ventral-derived blastemas. When these molecules were experimentally supplied to blastemas missing dorsal or ventral cells, *Shh* expression was restored – along with the ability to regenerate limb structures.

Together, these experiments reveal a previously unrecognized hierarchy in limb regeneration. Signals from the dorsal and ventral sides of the limb do not directly specify limb pattern themselves; instead, they create the conditions necessary for *Shh* expression in posterior cells, enabling the well-known anterior-posterior signaling that drives limb outgrowth and pattern formation (Figure 1).

**Figure 1.**
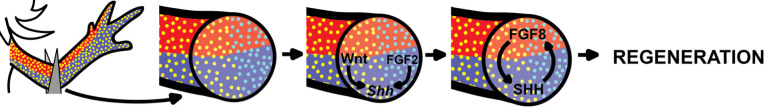
Signaling events and limb regeneration in axolotls. When an axolotl limb is amputated (left), cells in the anterior (red), posterior (purple), dorsal (yellow dots), and ventral (pale blue dots) sides of the limb contribute to the regenerative blastema. Molecular signals from both the dorsal and ventral cells (Wnt and FGF2) are required to activate the expression of the key patterning signal from the posterior cells. Once the gene *Shh* is expressed, it can generate a feedback loop with anterior cells that express FGF8 to regenerate a structurally complete limb structure.

While this study represents an important step forward in efforts to understand how axolotl limb cells use positional memories to generate the signals needed to organize the regenerating tissue, key questions remain. What makes injured axolotl limb cells capable of communicating and responding to organizational cues? And how do limb cells modulate these cues to regenerate only the missing structures, rather than duplicating the entire limb?

Recent studies have shown that retinoic acid signaling shifts the starting location of the blueprint based on proximodistal position (Duerr et al., 2025). From work on embryonic limb buds, we know that anterior-posterior and dorsal-ventral signals interact with proximodistal signals (Delgado and Torres, 2017), so interplay in the context of regeneration is likely. Still, whether these interactions occur in the regenerating environment and whether proximodistal signals are essential for – or dependent on – anterior-posterior and dorsal-ventral signaling remains unknown.

Finally, while we humans cannot regenerate our limbs, we do retain the key basic ingredient that salamander limbs use – positional memory. We do not yet know why injured human limb cells are unable to use these memories, as axolotl cells do, to recreate missing limb structures. However, these memories appear to play important roles in adult tissue maintenance in mammals (Chang et al., 2002; Rinn et al., 2008) and could even contribute to the success of skeletal tissue engraftments (Song et al., 2024). The goal of regenerative biology is to understand, at a mechanistic level, how axolotl limb cells interpret injury and positional information, and to determine why mammalian cells fail to do the same. By defining how positional memories from all sides of the limb converge to activate key organizing signals, this work offers a conceptual roadmap for identifying which regenerative instructions are absent, or inaccessible, in human tissues, and where future therapeutic strategies might intervene to restore them.
